# Reevaluation of the *BRCA2* truncating allele c.9976A > T (p.Lys3326Ter) in a familial breast cancer context

**DOI:** 10.1038/srep14800

**Published:** 2015-10-12

**Authors:** Ella R. Thompson, Kylie L. Gorringe, Simone M. Rowley, Na Li, Simone McInerny, Michelle W. Wong-Brown, Lisa Devereux, Jason Li, Lifepool Investigators, Ian G. Campbell, Lisa Devereux, John Hopper, Vicki Pridmore, Anne Kavanagh, Gillian Mitchell, Bruce Mann, Stephen Fox, Alison H. Trainer, Gillian Mitchell, Rodney J. Scott, Paul A. James, Ian G. Campbell

**Affiliations:** 1 Cancer Genetics Laboratory, Research Division, Peter MacCallum Cancer Centre, East Melbourne, Victoria, Australia; 2 Department of Pathology University of Melbourne, Melbourne, Victoria, Australia; 3 Sir Peter MacCallum Department of Oncology, University of Melbourne, Melbourne, Victoria, Australia; 4 Cancer Biology Research Center, Tongji Hospital, Tongji Medical College, Huazhong University of Science and Technology, Wuhan, Hubei, China.; 5 Familial Cancer Centre, Peter MacCallum Cancer Centre, East Melbourne, Victoria, Australia; 6 Discipline of Medical Genetics and Centre for Information-Based Medicine, The University of Newcastle and Hunter Medical Research Institute, Newcastle, Australia; 7 Lifepool, Peter MacCallum Cancer Centre, East Melbourne, Victoria, Australia; 8 Bioinformatics Core Facility, Peter MacCallum Cancer Centre, East Melbourne, Victoria, Australia; 9 Division of Genetics, Hunter Area Pathology Service, Newcastle, Australia; 10 Centre for Epidemiology and Biostatistics, Melbourne School of Population and Global Health, The University of Melbourne, Parkville, Australia; 11 BreastScreen Victoria, Carlton South, Victoria, Australia; 12 Melbourne School of Population Health, University of Melbourne, Parkville, Victoria; 13 The Royal Melbourne and Royal Women’s Hospitals, Parkville, Victoria, Australia; 14 Department of Pathology, Peter MacCallum Cancer Centre, East Melbourne, Victoria, Australia

## Abstract

The breast cancer predisposition gene, *BRCA2*, has a large number of genetic variants of unknown effect. The variant rs11571833, an A > T transversion in the final exon of the gene that leads to the creation of a stop codon 93 amino acids early (K3326*), is reported as a neutral polymorphism but there is some evidence to suggest an association with an increased risk of breast cancer. We assessed whether this variant was enriched in a cohort of breast cancer cases ascertained through familial cancer clinics compared to population-based non-cancer controls using a targeted sequencing approach. We identified the variant in 66/2634 (2.5%) cases and 33/1996 (1.65%) controls, indicating an enrichment in the breast cancer cases (p = 0.047, OR 1.53, 95% CI 1.00–2.34). This data is consistent with recent iCOGs data suggesting that this variant is not neutral with respect to breast cancer risk. rs11571833 may need to be included in SNP panels for evaluating breast cancer risk.

*BRCA2* was established as a high-penetrance breast cancer predisposition gene following its identification in 1995^1^,^2^. Hundreds of pathogenic mutations have been described that lead to a 55% risk of breast cancer by 70 years of age^3^. However, large numbers of variants in *BRCA2* are still of unknown clinical significance. For the most part these are missense variants, but the 3′ end of the gene can be affected by truncating mutations that appear to have little impact on breast cancer risk or protein function. One such variant is the c.9976A > T transversion (rs11571833), which leads to a nonsense mutation (K3326*) and loss of the final 93 amino acids. This variant was first assumed to be pathogenic^4^, but after subsequent observation in non-cancer controls^5^,^6^ it was reevaluated to be non-pathogenic and many investigators and clinical laboratories no longer reported its incidence. The prevalence of this variation in the normal population certainly supported the idea that it is not a high-penetrance breast cancer predisposition variant, as did its failure to fully segregate with disease in multi-case families^5^. In addition, *in vitro* functional data failed to find a difference from wild-type of K3326* with respect to cell viability, protein localization, homologous recombination repair and response to DNA damaging agents^7^,^8^.

Nonetheless, one study reported an enrichment of rs11571833 in multi-case pancreatic cancer families^9^. A relatively small SNP association study found a positive (OR 1.36), but non-significant association of the variant with bilateral breast cancer^10^, and the iCOGs analysis found an increased risk of breast cancer associated with this variant^11^. A complicating factor in attributing breast cancer risk to the rs11571833 variant is that it is in linkage dis-equilibrium with several non-coding variants (e.g. IVS 16-2A > G) and one class 5 pathogenic mutation (c.6503delTT)^5^,^12^,^13^. The c.6503delTT mutation is much rarer than rs11571833 but is almost universally observed in tandem with rs11571833. Thus, a predisposition signal for breast cancer could be derived from a strong effect of the much rarer pathogenic variant, rather than a small effect from the relatively common rs11571833.

In this study we re-evaluate the role of rs11571833 in breast and ovarian cancer predisposition using a panel mutation screen including all coding regions and intron/exon boundaries of *BRCA2*, in 2634 breast and/or ovarian cancer cases attending familial cancer clinics in Australia in comparison with ~2000 population-based non-cancer controls.

## Results

As part of a panel screening approach in breast cancer cases ascertained through familial cancer clinics in Australia, we evaluated the incidence of the *BRCA2* variant rs11571833 in 2634 cases and 1996 controls, which had previously been filtered to remove cases with pathogenic mutations in *BRCA1* and *BRCA2*, including the *BRCA2* c.6503delTT pathogenic mutation linked to rs11571833 (seen in one control). We detected rs11571833 in 66 cases (2.5% carrier frequency) and 33 controls (1.65%) (p = 0.047, Pearson chi squared, OR 1.53, 95% CI 1.00–2.34).

### Cases

The carriers of the variant with a diagnosis of breast (n = 64) or ovarian cancer (n = 2) had a mean age of diagnosis of 43.2 ± 9.6 (range 28–67) (Supplementary Tables S1 and S2), no different to cases overall (45.5 ± 10.6). For seven cases carrying the variant no details of the family history was available but 43 cases recorded a family history of breast/ovarian cancer (75%) and 16 cases had a family history of only other cancer types. Fifteen cases also had either a personal history of, or first degree relative with, bowel cancer (25%) compared to 12% in non-carriers. This difference represented a significant enrichment for bowel cancer in carriers compared to non-carriers (p = 0.004, Pearson chi squared, OR 2.5, 95% CI 1.37–4.59). There was no difference between carriers and non-carriers for family history of lung or prostate cancer. More detailed analysis of the extended pedigree information available for the Variants in Practice (ViP) study families found the average number of first or second degree relatives with breast cancer and/or first degree relatives with ovarian cancer was 1.45 in non-carriers and 1.21 in carriers.

### Controls

For the controls, 14/33 (42%) carriers reported having a first or second degree relative with breast/ovarian cancer, and 11 with a history of only other cancer types in first degree relatives (Supplementary Table S3). This was not different to non-carrier controls, where the proportion of women who reported at least one relative with breast or ovarian cancer was also 42%. When considering the strength of the family history, the average number of first and second degree relatives with breast cancer and/or first degree relatives with ovarian cancer was 0.58 in the non-carrier Lifepool controls (95% CI 0.55–0.62) and 0.70 in the carrier controls (95% CI 0.33–1.06). The number of carriers is too small to confidently identify a difference in the incidence of cancer in these families. There was no difference in the incidence of prostate or bowel cancer in the families of carriers and non-carriers (Table 1). The average age of the Lifepool carriers was 61.1 ± 10.1 (range 47–81), no different to the cohort overall (58.84 ± 9.9 years, range 19–91).

One control individual was homozygous for rs11571833, which was confirmed by Sanger sequencing (data not shown). This individual was 49 years of age and had not developed cancer of any type, although her mother had two breast cancers aged approximately 60 and 70. The control individual was diagnosed with an unspecified benign condition aged 41.

### Review of existing data

We searched the literature using the terms “K3326X” and “rs1157833” in Google Scholar, and identified 23 studies of breast and/or ovarian cancer that reported the number of cases with the variant (Supplementary Table S4). K3326* was identified in 107/6038 cases (1.8% carrier frequency). In addition, there were studies that found enrichment of the variant in pancreatic cancer (18/511 cases, 3.5%, 4 studies), lung cancer (218/9506, 2.3%, 2 studies), esophageal cancer (27/766, 3.5%, 2 studies) and squamous cell head and neck cancer (minor allele frequency 0.013 in cases, 1 study). The combined controls from these studies gives a frequency of 236/18,187 (1.3%). In other available control populations, rs1157833 is present at individual frequencies of 1.3% (Exome Variant Server, http://evs.gs.washington.edu/EVS/, Oct. 2014) and 1.1% (1000Genomes^14^). It is present in the Exome Aggregation Consortium, a collection of >61,000 cancer and non-cancer exomes, at 1.4% (http://exac.broadinstitute.org, Dec 2014).

There have also been a number of genome-wide association studies (GWAS) reporting on this variant: one US Caucasian study (OR 1.03, 95% CI not reported, p = 0.89^15^) and one US predominantly African-American study (OR 0.71, 95% CI 0.14–3.59^16^) found no association with breast cancer, however a European meta-analysis (OR 1.39, 95% CI 1.13–1.71^11^) and the subsequent overall iCOGS analysis (OR 1.26, 95% CI 1.14–1.39) were both significant for breast cancer. The Asian subset of iCOGS was not significant, despite an OR of 2.21 (95% CI 0.7–6.97^17^). A subset analysis of triple-negative breast cancer cases was significant (OR 1.44, 95% CI 1.05–1.96^18^). In all of these studies the majority of cases included were not pre-screened for pathogenic *BRCA2* mutations so the possible effect of linkage disequilibrium with the 6503delTT or other mutations on the reported OR must be considered. There was no genome-wide level association with ovarian cancer, either in iCOGs or an earlier study where 2.5% of cases and 2% of controls carried the variant^19^. A recent meta-analysis of prostate cancer GWAS was also not significant at a genome-wide level^20^. The significance of this variant in GWAS analysis of other cancer types has not been reported, however the SNP is not present on the commonly used Affymetrix or Illumina genotyping arrays, thus it may not have been represented.

## Discussion

The designation of the K3326* truncating variant in exon 27 of *BRCA2* as a breast cancer predisposing allele has changed over time as new evidence has emerged. In this study we reevaluate this variant and provide evidence that it may be associated with a low to moderate increase in the risk of disease. Key differences with this and other studies include our capacity to simultaneously detect the associated high-penetrance c.6503delTT variant and exclude this from both cases and controls, allowing assessment of the effect of the more common truncating variant alone, in contrast to GWAS studies where an increase in risk could be attributed to the presence of the high-risk variant. Another difference was our well-annotated group of population-based controls for which we were able to obtain family history information. This data suggested there may be a small increase in the strength of the family history of breast or ovarian cancer in carriers versus non-carriers, but we had limited power to detect a statistically significant difference. One caveat to our study was our inability to assess population substructure, however no population group studied to date has a carrier frequency as high as we have observed in the cases.

Functional studies of K3326* in cell lines have failed to demonstrate any effect on cell viability, homologous recombination (HR) or sensitivity to DNA damaging agents^7^,^8^. The C-terminus of BRCA2 contains a RAD51 binding domain that is important for the interaction of multimeric RAD51 with single-stranded DNA. A key serine residue for this process (S3291) is close to the truncating mutation, and a small protein sequence incorporating K3326 (amino acids 3265–3330) is capable of binding RAD51^21^. The interaction of the C-terminus with RAD51 appears to be less important for HR than it does for a more recently described function for BRCA2, protection of stalled replication forks from MRE11-mediated degradation^22^. Consequently, the assays previously used to evaluate the effect of K3326* may not have been ideally suited to assess the most significant impact of the variant on normal BRCA2 function. In addition, most assays used have employed an overexpression system for complementing BRCA2-null cells. Such systems with excess BRCA2 present may not be sensitive enough to detect subtle inefficiencies imparted by partial deletions of a functional domain, such as might be expected to lead to low-penetrance predisposition to cancer.

In conclusion, our results indicate that although K3326* does not have the same clinical significance as other *BRCA2* truncating mutations located more 5′ in the gene, its impact on modifying breast and/or ovarian cancer is not negligible. This variant may need to be included in panels testing all low-penetrance predisposition SNPs once robust risk estimates are obtained.

## Methods

### Case-control cohorts

Breast-cancer affected, unrelated index cases were ascertained from two locations: 998 individuals from the Hunter Area Pathology Service (“HAPS”), Newcastle, Australia^23^ and 1643 from the ‘Variants in Practice’ (ViP) Study recruited from Familial Cancer Centres (FCCs) in Victoria, Australia. All cases were either diagnosed at a young age (<50 or pre-menopausal) and/or had a significant family history of breast and/or ovarian cancer that fulfilled local eligibility criteria for clinical genetic testing (previously described in Sawyer *et al*.^24^). Clinical genetic testing, involving screening of all exons of *BRCA1* and *BRCA2*, was previously performed in all cases and individuals with pathogenic mutations were not included in this study.

The control cohort was 1996 participants in the LifePool study (www.lifepool.org) who were cancer-free at the time of enrollment. This study aimed to recruit women over 40 years in age through the population-based mammographic screening program in the state of Victoria, Australia (BreastScreen Victoria). All participants provided epidemiological and mammographic data and were followed up through the Victorian Cancer Registry to detect cancer incidence. The average age of the participants was 58.84 ± 9.9 years (range 19–91).

All cases and controls provided informed consent for genetic analysis of their germline DNA for research purposes and publication. This study was approved by the Human Research Ethics Committees at each of the participating centres, including the Peter MacCallum Cancer Centre Human Research Ethics Committee. This study was carried out in accordance with all relevant regulations and guidelines.

### Panel-based variant screening

Germline DNA (225 ng) mostly from blood (all cases and >97% of controls) or saliva (2.6% of controls) was analysed using two methods. A subset of ViP study cases (n = 646) were specifically genotyped for the rs11571833 variant using a Fluidigm sequencing assay^25^. Full *BRCA2* mutation status for these 646 cases was accessed from clinical diagnostic sequencing data. The remaining cases and all controls were tested using a custom gene panel that included all exons of *BRCA1* and *BRCA2*. Exons were amplified using the HaloPlex Targeted Enrichment Assay (Agilent Technologies) and an Agilent Bravo Automated Liquid Handling System according to the manufacturer’s protocol. Indexed, 96-sample pooled libraries were analysed on a HiSeq2500 Genome Analyzer (Illumina) to generate paired-end 100 or 150 bp sequencing reads. Sequencing alignment was performed using BWA^26^, and base quality score recalibration and indel realignment was performed by GATK^27^. Variant calling was undertaken using GATK Unified Genotyper v2.4^28^. Variants were determined by reference to NM_000059.3 (GenBank) according to HGVS guidelines (www.hgvs.org/mutnomen).

## Additional Information

**How to cite this article**: Thompson, E. R. *et al*. Reevaluation of the *BRCA2* truncating allele c.9976A > T (p.Lys3326Ter) in a familial breast cancer context. *Sci. Rep*. **5**, 14800; doi: 10.1038/srep14800 (2015).

## Supplementary Material

Supplementary Information

Supplementary Information

## Figures and Tables

**Table 1:** Family histories of cases/controls by rs11571833 status.

Family History type	HAPS carriers	FCC carriers	FCC non-carriers	LP carriers	LP non-carriers
Breast/ovarian 1st degree	8 (47%)	22 (52%)	895 (57%)	8 (24%)	437 (22%)
Breast 2nd degree	7 (41%)	17 (40%)	733 (46.5%)	11 (33%)	552 (28%)
Breast/Ovarian 1^st^ and/or Breast 2^nd^ degree	12 (71%)	31 (74%)	1210 (77%)	14 (42%)	832 (42%)
Number 1^st^ /2^nd^ degree with Br/Ov	NA	1.21	1.45	0.70	0.58
Other cancer only, 1st degree	5 (29%)	11 (26%)	352 (22%)	11 (33%)	788 (40%)
Bowel cancer 1st degree	3 (18%)	11 (26%)	189 (12%)	4 (12%)	287 (15%)
Other cancer only, 2nd degree^1^	3 (18%)	19 (45%)	502 (31.8%)	11 (33%)	635 (37%)
No. with family history recorded	17	42	1578	33	1956
No. without family history recorded	7	0	16	0	7

^1^Denominator different for Lifepool as some 2^nd^ degree family history not known.
