# Supplementary material for: Reevaluation of the BRCA2 truncating allele c.9976A > T (p.Lys3326Ter) in a familial breast cancer context
Source: Sci Rep. 2015 Oct 12;5:14800. doi: 10.1038/srep14800 (PMC4601142; doi:10.1038/srep14800)
Supplement: Supplementary Information [file srep14800-s1.pdf]

Reevaluation of the *BRCA2* truncating allele c.9976A>T (p.Lys3326Ter) in a familial breast cancer context.

Ella R Thompson[1], Kylie L Gorringe[1,2,3], Simone M Rowley[1], Na Li[1,4], Simone McInerny[5], Michelle W Wong-Brown[6], Lisa Devereux[1,7], Jason Li[8], Lifepool Investigators[7], Alison H Trainer[1,5], Gillian Mitchell[5], Rodney J Scott[6,9], Paul A James[2,3,5], Ian G Campbell[1,2,3]

[1]Cancer Genetics Laboratory, Peter MacCallum Cancer Centre, East Melbourne, Victoria, Australia. [2]Department of Pathology and [3]Sir Peter MacCallum Department of Oncology, University of Melbourne, Melbourne, Victoria, Australia. [4]Cancer Biology Research Center, Tongji Hospital, Tongji Medical College, Huazhong University of Science and Technology, Wuhan, Hubei, China. [5]Familial Cancer Centre, Peter MacCallum Cancer Centre, East Melbourne, Victoria, Australia. [6]Discipline of Medical Genetics and Centre for Information-Based Medicine, The University of Newcastle and Hunter Medical Research Institute, Newcastle, Australia. [7]Lifepool, Peter MacCallum Cancer Centre, East Melbourne, Victoria, Australia. [8]Bioinformatics Core Facility, Peter MacCallum Cancer Centre, East Melbourne, Victoria, Australia. [9]Division of Genetics, Hunter Area Pathology Service, Newcastle, Australia.

Supplementary Tables 1-3. Details of carriers.

1. HAPS cases with rs1157833

Case	Age of diagnosis	Cancer type	Family history 1st degree	Family history 2nd/3rd degree
HAPS-100240	48		Breast	AML, ovarian
HAPS-100265	43		Breast	Breast
HAPS-100356	67		Breast	Breast, ovarian, pancreatic
HAPS-100468	57			Ovarian, colorectal
HAPS-100556	43	Breast	-	
HAPS-100699				
HAPS-101410	53		Breast, colorectal	Prostate, endometrial
HAPS-101870			-	
HAPS-102557			Breast	
HAPS-110368				
HAPS-111476			Strong family history	
HAPS-112654	62	Breast	Colorectal, prostate, lymphoma	
HAPS-113784			-	
HAPS-113873	60	Breast	Colorectal, Oesophageal	Breast
HAPS-113981	57	Ovarian		Breast
HAPS-120274	56, 63			Breast
HAPS-120625	31, 54	Bilateral breast	-	
HAPS-120996	55		Cervical, prostate, bladder	Lung, colorectal, cervical
HAPS-121334	36, 48	Bilateral breast	Glioblastoma, Cancer NOS	Breast
HAPS-80021	36	Breast		Ovarian
HAPS-81700	51, 60	Bilateral breast	Breast	
HAPS-90359	57		Prostate, breast	Breast
HAPS-90833	46	Ovarian	Lung	Colorectal
HAPS-92185				

2. FCC cases with rs1157833

Case	Age at diagnosis	Cancer Type	Family history 1st degree	Family history 2nd degree
FCC-1693-000	42	Breast	Breast, other	Other
FCC-1858-000	28, 28	Breast	Bowel, other x3	Other
FCC-2086-000	66	Breast	Breast x2, Bowel	Lung
FCC-2089-000	38	Breast	Other	Breast, Lung, Other
FCC-210-000	38	Breast	Breast/Bowel (same person)	Breast
FCC-2165-000	36	Breast	.	Lung
FCC-2384-000	40,46	Breast	Breast/Bowel (same person)	Bowel x2, Other x3
FCC-2432-000	56	Breast	Breast, Ovarian, Prostate	Breast
FCC-2626-000	42	Breast	Bowel, other	Breast, Bowel x2, Other x4
FCC-280-000	53, 54	Breast, Ovary	Breast, Prostate	Lung, Other
FCC-2838-000	47,53	Breast	Otherx3	Prostate
FCC-2876-000	38	Breast	.	Breast, Lung
FCC-3083-000	36	Breast	.	Breast, Bowel, Prostate, Other x3
FCC-3119-000	32	Breast	.	Ovarian, Other
FCC-316-000	42	Breast	Breast x2	Breast x3
FCC-3192-000	52	Breast	Breast, Other	Breast

FCC-3275-000	50,58, unk	Breast x2, Bowel	Other x2	Breast x2, Bowel x2, Prostate, Other x3
FCC-3278-000	62	Breast	Prostate	Lung
FCC-3294-000	33	Breast	.	Other x2
FCC-3364-000	27	Breast	.	Other x2
FCC-3565-000	28	Breast	Other	Breast x3, Lung, Other
FCC-3870-000	29	Breast	Other	Other x2
FCC-907-000	48	Breast	Breast, other	Breast, Bowel, Other x2
FCC-581-000	52	Breast	Breast, Bowel	.
FCC-551-000	52	Breast	Breast	Other x2
FCC-1358-000	43	Breast	Breast x2, Bowel	Other
FCC-260-000	42	Breast	Breast	Lung, Other
FCC-1039-000	49	Breast	Breast, Prostate	Bowel
FCC-526-000	54	Breast	Breast, Bowel	Breast, Bowel
FCC-616-000	37	Breast	.	Bowel, Other x3
FCC-1016-000	33	Breast	Breast	Lung, Bowel, Other x2
FCC-1063-000	48	Breast	Ovarian, Bowel	Breast x2
FCC-624-000	40	Breast	Breast, Bowel, Prostate	Prostate x2, Other x2
FCC-300-000	44	Breast	Other	Prostate, Other x3
FCC-1114-000	44	Breast	Breast	.
FCC-589-000	38	Breast	Other	Breast/Lung, Lung
FCC-1515-000	42	Breast	Breast x2	Breast
FCC-186-000	56	Breast	Breast x2, Bowel x2	.
FCC-656-000	41	Breast	Other	.
FCC-1115-000	44	Breast	.	Breast, Other
FCC-1268-000	61	Breast	Breast, Other	Ovarian
FCC-2453-000	34	Breast	.	Breast, Other

3. Controls with rs1157833

Controls	Age	Family history 1st degree	Family history 2nd degree
LP-12048272	49	Colorectal	Other
LP-12052032	55	N	**Breast**, Other
LP-12052841	50	N	N
LP-12053358	51	**Breast**, Colorectal, Prostate	**Breast**
LP-12053724	68	Other	**Breast**
LP-13000214	72	N	N
LP-13027406	55	N	Other
LP-13036592 *homoz	49	**Breast**	Other
LP-13041356	81	Other	Other
LP-13090810	52	**Ovarian**, Prostate	NA
LP-13100510	49	**Breast**	NA
LP-11000316	76	Testicular, Melanoma	Other
LP-11000415	47	**Breast**	**Breast**, Other
LP-12003580	80	Colorectal	N

LP-11000799	71	**Breast**	**Breast**, Other
LP-12005688	64	Lung, Other	N
LP-12003677	63	Colorectal	Other
LP-12000264	72	N	Other
LP-12006186	59	N	N
LP-12010562	51	**Ovarian**	**Breast**
LP-12007674	62	Lung, Other	Other
LP-12009531	71	**Breast**	**Breast**
LP-12011226	65	N	**Breast**, Other
LP-12007515	49	N	Other
LP-12009764	66	N	N
LP-12010367	76	Prostate	Other
LP-11000229	66	Prostate	N
LP-10000188	56	N	**Breast**
LP-12031819	65	N	**Breast,** Other
LP-13039185	52	N	Colorectal, Other
LP-13081169	51	N	Other
LP-13067508	62	Other	**Breast**
LP-13231021	59	N	N
